# Integrating public health and primary care: a framework for seamless collaboration

**DOI:** 10.3399/BJGPO.2024.0096

**Published:** 2024-11-27

**Authors:** Luke N Allen, Bernd Rechel, Dan Alton, Luisa M Pettigrew, Martin McKee, Andrew David Pinto, Josephine Exley, Eleanor Turner-Moss, Kathrin Thomas, Jacqueline Mallender, Dheepa Rajan, Toni Dedeu, Simon Bailey, Nicholas Goodwin

**Affiliations:** 1 Global Primary Care and Future Health Systems, University of Oxford, England, UK; 2 University of New South Wales, Sydney, Australia; 3 European Observatory on Health Systems and Policies, London School of Hygiene & Tropical Medicine, London, UK; 4 NHS England and Buckinghamshire, Oxfordshire and Berkshire West NHS Integrated Care Board, Oxford, UK; 5 Health Services Research and Policy, London School of Hygiene & Tropical Medicine, London, UK; 6 Upstream Lab, University of Toronto, Ontario, Canada; 7 Faculty of Public Health group on Public Health & Primary Care, London, UK; 8 Economics By Design, London, UK; 9 European Observatory on Health Systems and Policies, Brussels, Belgium; 10 World Health Organization, Regional Office for Europe, WHO European Centre for Primary Health Care, Almaty, Kazakhstan; 11 Central Coast Research Institute for Integrated Care, University of Newcastle and Central Coast Local Health District, Gosford, Australia

**Keywords:** public health, primary care, health policy

Integration between public health and primary care is rising on the health policy agenda but the terms and concepts involved can be confusing. This article reviews the relevant literature and presents a new framework to help policymakers think through what they are aiming to achieve and why. We unpack different degrees and types of integration and show how they fit together. We argue that the merger of public health and primary care into a single entity with one aim, budget, and one multidisciplinary team isn’t necessarily the desired end-point for most health systems. Seamless collaboration will likely improve patient and health system outcomes, save resources, and improve population outcomes. We recommend that efforts to foster better collaboration should take an activity-based approach, promoting alignment of teams, training, budgets, values, and culture around specific tasks, and in proportion to need.

## Introduction

The delivery of ‘primary care and public health as the core of integrated health services’ is one of the foundational components of Primary Health Care (PHC), as defined in the Alma-Ata and Astana Declarations, and in the World Health Organization (WHO) Vision for PHC in the 21st century.^1–3^ The COVID-19 pandemic required many rapid adjustments to how public health and primary care services were delivered, fostering improved collaboration and integration across international health systems.^4–7^ The pandemic also exposed critical areas of misalignment, miscommunication, and missed opportunities.^8–10^ Both sets of experiences seem to have raised the priority of strengthening integration between public health and primary care.

Public health interventions have often operated in silos that are separate from clinical care, despite common objectives including the promotion of health and healthy environments; preventing illness; treating and managing disease; and improving quality of life. Without better integration the whole of the health and care system risks suffering from suboptimal outcomes, with patients and carers missing out on holistic care, a workforce operating in silos that is unable to tackle the complex issues in front of them, unnecessary utilisation of services or medications and, potentially, a threat to the longer-term financial and operational sustainability of the system itself.^8,11–13^

While few would argue against greater integration, there is often little agreement as to what it means. Indeed, the idea is so poorly defined that its advocates often end up talking at cross-purposes. Existing frameworks tend to present Venn diagrams that illustrate overlapping functions, roles, and competencies, but stop before they define the principles and actions that should underpin closer working or address the many structural and ideological barriers to doing so.^12–19^

In this article we offer definitions of primary care, public health, and integration. We then set out a framework for thinking about closer working at different levels across the health system. Our aim is to equip those working at the interface of public health and primary care with an overview of the core concepts and to provide a framework for orientation along the spectrum from isolation to merger.

### What do we mean by 'public health' and 'primary care'?

Public health is often defined as *‘the science and art of preventing disease, prolonging life and promoting health through the organised efforts of society'*.^20^ The essential functions of public health have been described in various frameworks,^21–24^ which the WHO ‘PHC Primer’ has condensed into five core functions that are specifically relevant to a PHC approach and closely linked to primary care service delivery. These are: health protection, health promotion, disease prevention, surveillance, and emergency preparedness.^3^

Primary care is a community-based service delivery platform that is commonly defined in terms of five core characteristics (Table 1).^19,25,26^

**Table 1.** Core characteristics of primary care

Core characteristics of primary care
First contact - primary care is the first part of the health system that patients contact for non-emergency health issues, and the front door to the wider health system
Continuity - the same clinician, team, or organisation sees patients regularly over the course of their lives and for most of their health conditions
Comprehensiveness - primary care teams provide care for the vast majority of health needs in the community with services spanning health promotion, prevention, diagnosis, treatment, rehabilitation, and palliation
Coordination - primary care teams take responsibility for arranging referrals and investigations to other parts of the health and care system when necessary, collating results and correspondence, and providing a holistic overview of each person’s care.
Person-centredness - primary care teams provide holistic biopsychosocial care that accounts for people’s and their communities’ needs, preferences, and expectations

Primary care clinicians — often working in general practice or family medicine teams — play the lead role in delivering comprehensive, coordinated, continuous, and person-centred care in many settings, usually working in concert with other community-based service providers. Many general practice and family medicine training schemes stress the dual role of primary care in caring for individual patients alongside holding a (varying) degree of responsibility for the health of the local community, or at least those on a list of patients registered with them (empanelment), and sometimes paid according to the number of registered patients (capitation).

Primary care services often get mixed up with the broader term ‘Primary Health Care’: a wider concept that describes a whole-of-society approach to health based on multisectoral action, community engagement, and integrated health systems.^3^ ^27^At the other end of the spectrum, in many countries the term ‘primary care’ is used to describe any form of first-contact, community-based service, even if it does not offer continuous, comprehensive, coordinated, or person-centred care. For the purposes of this article, we use the WHO and Starfield definition of primary care, defined by the five core characteristics listed in Table 1.

### How do public health and primary care differ?

Public health is primarily concerned with levels and distributions of health outcomes in a defined population, whereas primary care services tend to focus on the needs of individuals at the same time as holding responsibility for health outcomes and addressing inequalities in their local community. While primary care teams do not routinely lead on health protection, their work feeds into surveillance and monitoring, population health management, and the delivery of individual and community-level health promotion and disease prevention activities, which touch on core public health functions. Both public health and primary care functions require some degree of community engagement although, in practice, it may be more frequently and systematically carried out within public health.

Much of the drive to integrate public health and primary care stems from the desire to optimise coordination between the two disciplines in these overlapping areas to maximise health outcomes, tackle inequalities, and reduce inefficiencies. However, different types of collaboration are required for different areas of work, and at the various micro (individual clinic/neighbourhood), meso (local network/regional) and macro (national) levels within the health system.

### What do we mean by 'integration'?

At its core, integration is about bringing different components together to form a seamless whole,^28^ however scholars have advanced a panoply of different concepts covering different degrees,^14,29,30^ types,^31,32^ and processes^31,33^ of integration (discussed fully in the Appendix). Table 2 presents five degrees of implementation and their professional, organisational, and functional characteristics, building on the work of Nolte and McKee, Contandripoulos, and Ham and Gerada.^31,32,34^

**Table 2.** Degree and type of integration

Degree	Type		
	*Professional*	*Organisational*	*Functional*
***Merger***	All staff are employed by the same entity and work in a single multidisciplinary team	There is a single organisation that delivers comprehensive services to a defined population	There is a single integrated system with unified plans, human resources, financial and information management, and quality improvement
***Collaboration***	Regular planned meetings, joint training, and possible co-location help relationships and shared understanding of roles to form	Shared governance and contractual arrangements, networks, and alliances all facilitate joint organisational working for specific areas of collaboration	Joint planning, information sharing, and coordinated financial & resource management enable seamless collaboration for set activities
***Cooperation***	Teams only interact on an ad-hoc basis, and are unlikely to have trained together	Broad arrangements may be in place to govern sharing of information and resources between organisations	Ad-hoc activities are coordinated. Planning, resources, and information can be shared, but often with friction at the interface
***Overlap***	Teams may be aware of one another but rarely meet. They may not know how to initiate contact	Despite holding responsibility for shared issues, both organisations operate independently	Activities are not coordinated in any way and there may be some duplication of work
***Isolation***	Teams never meet, don’t know what the other is doing, and don’t know how to contact each other	Contracting and governance arrangements entrench separation. Primary care focuses exclusively on reactive care for individuals	Activities are not coordinated in any way and organisations may be competing for resources

Many of these concepts revolve around communication. Effective communication of information is recognised as an essential component of successful integrated care programs.^35,36^ Specifically, such evidence points to the importance of systems having the capacity to communicate data and information across the system, manifest in key capabilities such as patient identifiers, shared care records, and effective use of such data in decision-making and care delivery. Indeed, the same evidence indicates that effective communication is likely the most important factor that enables care teams to work constructively together and co-ordinate care with and around people’s needs - more so than changes to organisational form, governance, or financial incentive structures that seek the same ends.

**Figure 1.**
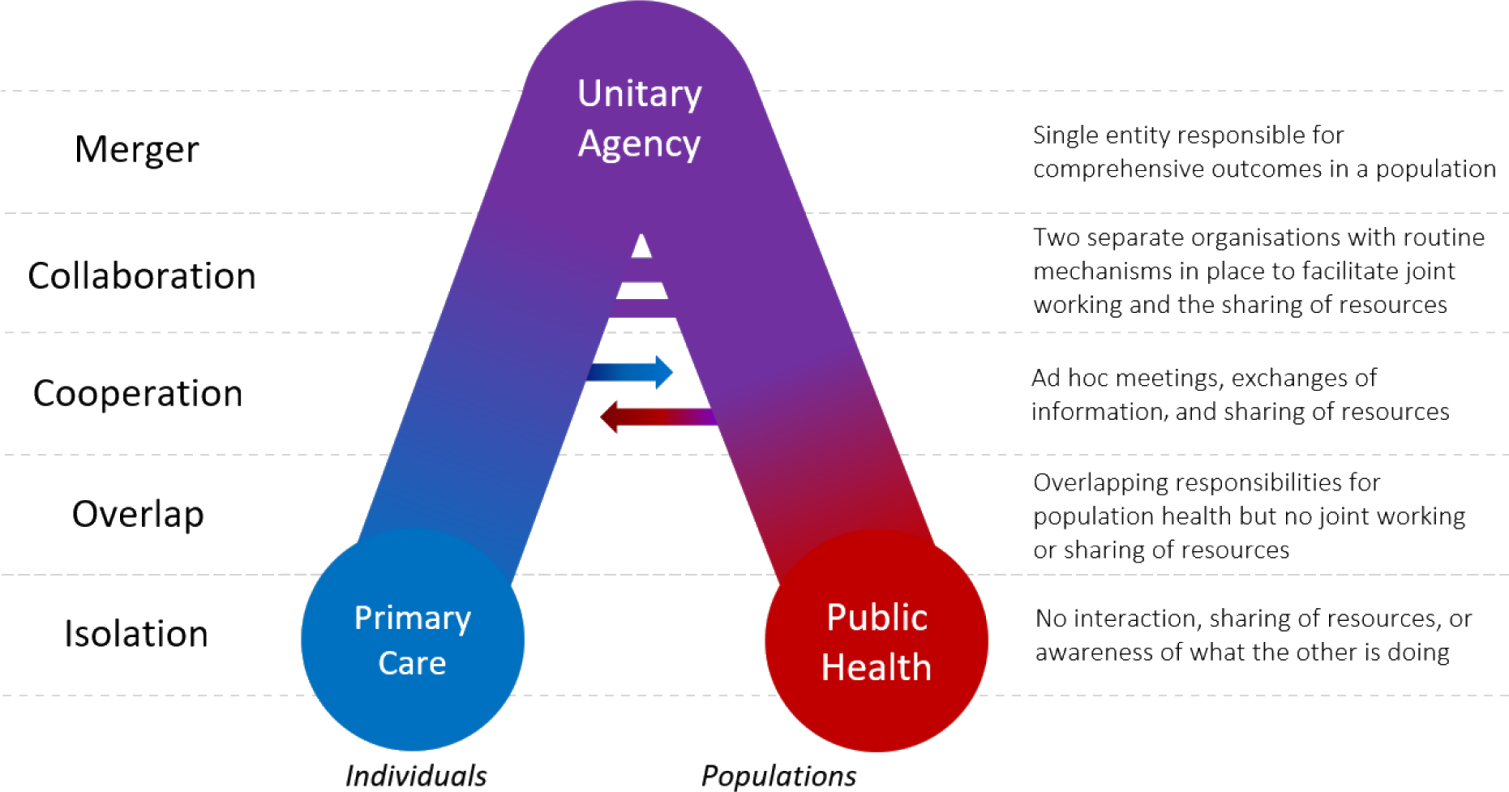
Framework for public health and primary care integration

Figure 1 reconstitutes the degrees of integration as a conceptual diagram, plotting primary care organisations on the horizontal axis in terms of the extent to which they engage with populations. The rest of this section unpacks each degree in greater detail.

#### Isolation

This is often the starting point. Public health teams operate without engaging with primary care in any way and primary care delivers individual-level interventions aimed largely at reactively managing symptoms and illnesses that have already arisen. Primary care teams may have no or little public health training, no dedicated time to think about population health, no means to address local social determinants, and no financial or other incentives to keep people well or prevent disease. Unfortunately, it is relatively common for public health and primary care teams to operate in complete isolation, with no awareness of what the other is doing or even how to go about contacting a relevant counterpart should a need arise. An example of this type of working is the model that was common in many former Soviet countries, where primary care was provided by 'specialists' with expertise in a focused area and a narrow clinical focus, working in polyclinics, while public health (mainly infectious disease control) was organised in parallel vertical systems.^37^ Most countries in this part of Europe have moved beyond this model, to greater or lesser extents.

#### Overlap

Primary care takes the first steps towards engaging with public health by assuming responsibility for the delivery of proactive prevention and health promotion activities to their patients. Empanelment, capitation, community engagement, and electronic health records are the essential foundations for this type of work. Whilst primary care and public health share overlapping responsibilities, members of staff from the two groups do not train or meet together, communicate, exchange information, or share resources. There may be some vague awareness of what the other group is doing. For instance, primary care teams may have heard about a local public health campaign around stopping smoking, or, because the same communities are being ‘engaged’, information might informally pass through the community channel. However, neither team is meaningfully in touch with the other. There is no functional integration around key activities, or shared governance and contracting which can lead to inefficient duplication of services like cervical cancer screening. This approach is the baseline for a large part of primary care provision around the world today,^38^ including across much of the USA.^7,39^

#### Cooperation

Public health and primary care still operate largely independently of each other but reach out to request information or collaborate on an *ad hoc* basis. Examples might include requests for primary care data on the prevalence of a certain condition, or joint working to deliver COVID-19 vaccinations. The two may share space, personnel, or resources for time-limited, one-off activities. However, these interactions can take a bit of time to get going as there are no established ways of working together, no focal points within each organisation, and no dedicated governance arrangements to orchestrate the secure exchange of data or resources. Canada’s Family Health Teams provide an example of this type of working.^12^

#### Collaboration

It is clear who to contact within each organisation, there are established pathways for requesting data, and there are routine information exchange mechanisms. There is a continuity of information— that is, staff within different organisations can seamlessly access each others’ data — even if both groups may use different information management systems. There are clearly defined roles and responsibilities in the context of routine coordination mechanisms, such as co-location of staff, community engagement undertaken jointly, or regularly scheduled meetings. Patient data are linked and used by public health analysts to support population health management, and primary care teams use this intelligence to offer tailored interventions for priority groups. Primary care teams refer notifiable cases to public health, and people identified with relevant health needs are referred to primary care teams by public health. Despite coordinated planning and working around well-defined activities, public health and primary care remain distinct entities with their own staff, training programmes, budgets, and lines of accountability. An example of this type of approach are the Health Promotion Centres that have been established in all Primary Health Care Centres in Slovenia.^40^

#### Merger

A single organisation assumes all responsibility for promoting health, preventing illness, and managing disease for a defined population, which includes delivering essential public health functions alongside treating individual patients. This unitary agency has a single budget and employs multidisciplinary teams. There is one vision, one shared set of overall objectives, and a cohesive culture. Some individual members of staff may spend most or even all of their time on either clinical primary care or public health-specific activities, but they are likely to have trained and socialised with members of other teams and share a common culture and approach. Such entities might encompass areas such as school and occupational health. El Salvador’s Territorial Community Teams exemplify many of these elements, as do some aspects of Cuba’s health system where multidisciplinary teams hold responsibility for identifying and meeting the combined public health and primary care needs of defined populations.^41^ The concept of Community Oriented Primary Care is also well aligned with the concept of merger.^42,43^

### How is the term ‘integration’ currently used in practice?

As we have highlighted, there are many concepts and terms in this space. However, when primary care and public health teams talk about ‘integrating’ the two disciplines in practice, they generally mean one of two things.

The first broad conceptualisation revolves around better integrating public health functions into everyday primary care practice; that is, arguing that health promotion and disease prevention should form part of the comprehensive care delivered in primary care. This does not necessarily entail substantial integration or input from public health. It does, however, require that primary care training, contracting, governance, and payment arrangements change to enable a focus on disease prevention, health promotion, and proactive ‘upstream’ action on the social determinants of health at the local population level. This might also include enhancing the epidemiology and public health training that primary care clinicians receive. This is best captured by the move from *isolation* to *overlap* in the degree of integration. It also requires that primary care organisations adopt public health norms, principles, and certain functions.

The second predominant conceptualisation is concerned with fostering greater collaboration between existing teams and/or organisations to achieve efficiency savings and improved health outcomes in areas where their remits overlap at the local, regional, or national level. Here the implicit focus is often on changes at the level of primary care clinics or local networks. An example would be the increasing global focus on population health management which is enabled by primary care practices collaborating with local public health teams around the sharing of patient data, segmentation, targeting, intervention design, outreach, and delivery of tailored interventions,^44^ grounded on the foundation of patient empanelment.^45^ This conceptualisation is best captured by the move from *overlap/cooperation* to *collaboration* via the establishment of dedicated governance arrangements, interoperable information systems and information sharing pathways, regular meetings, joint planning, and the routine sharing of people, places, and resources for joint activities; touching on all types and processes of integration.

We would argue that whilst both these conceptualisations have validity, the first (enabling primary care to have a stronger population level focus on health promotion and disease prevention) does not necessitate integration with public health teams or organisations, and the latter (deeper collaboration between the two) is often expressed as an indistinct aspiration.

### Where do we want to be?

As stated above, the ‘top’ of the framework — *merger* — is not necessarily the ideal destination for health systems at the macro-, meso-, or micro-level. There are also many areas within public health and primary care where there is no need to work together; for example, some purely clinical areas of primary care and in certain areas of health protection. Nevertheless, there are still myriad areas where organisations and teams are duplicating effort or labouring on tasks that would be easier and more effective with greater collaboration.

Goodwin and Fer have argued that public health remains peripheral to planning and purchasing of health and social care services and, as a result, public health interventions have often tended to operate in separate silos in the provision of health and social care.^46^ However, closer communication and integration between primary care and public health is foundational to the success of the so-called ‘fifth wave’ of public health, characterised by a focus on population health management, health promotion, and ill-health prevention (and following on from previous waves that focused on public works, medicine as science, the welfare state, and systems thinking).^47^ Table 3 outlines the structures that are being introduced in England to foster greater collaboration at the local level.

**Table 3.** The current English approach to encouraging collaboration at the local level

In England, the concepts of cooperation, collaboration and networking are being delivered through the creation of Integrated Neighbourhood Teams (INT) operating at a population level of around 30 000 to 50 000. At the macro level, ‘Integrated Care Systems’, a broad alliance of health and care providers who have a role in improving local health, care, and wellbeing, set the overall strategic direction and priorities for the region. Integrated Care Boards translate the strategic goals of ICSs into actionable plans, and then INTs are intended to operationalise the plans at the grassroots level, responding to the unique needs of local communities. INTs are generally based around groupings of general practices called ‘Primary Care Networks’ and are facilitated through a more formalised collaborative structure of local leadership created at ‘Place’ level, with Places encompassing populations of around 500 000. The concept allows, for example, grassroots local community involvement at the INT level as well as local government leadership involvement at Place and System level. This can be achieved without formal merging of organisational structures, but brings a team of leaders together at each level to deliver a more integrated approach to Primary Care and Public Health (among other objectives) which is tailored to local population needs and informed by local data.

We recommend that policymakers should take an activity-based approach to reform and support a move towards deeper integration in areas where the delivery of improved patient or system outcomes is dependent on greater alignment of teams, training, budgets, systems, values, and culture. The degree of reform to achieve integration should be proportionate to the requirements required by each specific activity. For instance, feeding data on influenza diagnosed in primary care to public health teams for seasonal surveillance depends on interoperable data exchange systems and governance arrangements but does not necessarily require regular staff meetings or pooled financial resources. In contrast, mature population health management depends on close collaboration, data exchange, shared analytics, and joint action, supported by contracting and governance arrangements. Policymakers could start by delineating the specific public health functions and primary care activities that require both public health and primary care input across the areas of health protection, health promotion, disease prevention, emergency preparedness, and surveillance and monitoring. For example, joint planning activities should be focused on areas like population health management, community engagement, and addressing the local social determinants of health where mandates overlap.^17^ Similarly, joint training would be most helpful when focused on shared competencies and activities that require collaborative working, such as planning health services, community engagement, addressing inequalities, or responding to disease outbreaks.

Our framework can be used to identify the most appropriate type and degree of collaboration for activities in each domain. We would argue that all primary care organisations should seek to transition from *isolation* to *overlap* by being resourced to assume a degree of responsibility for the health of local communities, including health prevention and health promotion work where this is not already the case. This requires attention to training, financing, workforce, and incentives, including contracting and payment models. Capitation can be used to link primary care providers with the wider population they serve, enhancing alignment with public health aims and principles. However, the prospective nature of capitation means that it is associated with a risk of under-provision when used in isolation.^48^ Blended models can ameliorate these risks, as seen in Canada and the UK where primary care capitation is supplemented with financial incentives (pay-for-performance) around key public health outcomes.^49^

Promoting public health and primary care collaboration would also be served by greater resourcing of joint learning networks to deepen relationships and support collaboration, as well as the sharing of perspectives and ideas. The UK Faculty of Public Health *Primary Care and Public Health Special Interest Group* is an example of this kind of voluntary network,^50^ and the Canterbury Clinical Network in New Zealand is a further example of a formal alliance of healthcare professionals, providers and system leaders.^51^

### Conclusion

The integration of public health and primary care is often talked about, but covers a multitude of partially overlapping concepts. In synthesising the available literature, we aimed to bring clarity and consistency to the terms. There is a need for operational, ‘on the ground’ relationships that enable public health strategies and teams to be seamlessly integrated into the work of primary care. Our framework builds upon other important work in this area and can be used as a starting point for policymakers and practitioners as they come together to discuss collaboration. We advocate for an activity-based approach to integration, seeking closer alignment of teams, organisations, and functions to the extent that joint objectives require. We stop short of discussing the drivers, enablers, and facilitators because these have been well covered in other publications. Future work could use our framework to benchmark the different degrees and dimensions of integration of contemporary public health and primary care practice. For now, policymakers and providers should take stock of where they are, consider where they want to be, and reflect on the specific types and processes of integration that are needed to deliver better value for their patients, populations, and health systems.
